# Dr. H. Warner Eggleston

**Published:** 1912-05

**Authors:** 


Dr. H. Warner Eggleston of Binghamton, a graduate of the University of Vermont, 1895, died April 11, 1912.

